# A rare case of paradoxical pulmonary embolism in spontaneous aortocaval fistula

**DOI:** 10.1259/bjrcr.20200183

**Published:** 2021-01-06

**Authors:** Valentina Vespro, Stefano Fusco, Anna Maria Ierardi, Viviana Grassi, Ilenia D’Alessio, Silvia Crespi, Maria Carmela Andrisani, Andrea Bellobuono, Santi Trimarchi, Gianpaolo Carrafiello

**Affiliations:** 1 Department of Radiology, Fondazione IRCCS Ca’ Granda Ospedale Maggiore Policlinico, University of Milan, Milan, Italy; 2 Postgraduate School of Diagnostic and Interventional Radiology, University of Milan, Milan, Italy; 3 Department of Vascular Surgery, Fondazione IRCCS Ca’ Granda Ospedale Maggiore Policlinico, University of Milan, Milan, Italy; 4 Postgraduate School of Vascular Surgery, University of Milan, Milan, Italy; 5 Department of Health Science, University of Milan, Milan, Italy

## Abstract

Aortocaval fistula (ACF) is a rare complication of abdominal aortic aneurysm (AAA), occurring in less than 1% of all AAAs.

Paradoxical embolism can rarely be associated with ACF, pulmonary embolism may originate from dislodgment of thrombotic material from the AAA in the inferior vena cava (IVC) through the ACF.

We report a case of a patient admitted to the emergency department with abdominal pain and shortness of breath who immediately underwent thoraco-abdominal CT. Imaging allowed a prompt pre-operative diagnosis of an ACF between an AAA and the IVC, also identifying CT signs of right heart overload and the presence of a paradoxical pulmonary embolism.

## Case presentation

A 57-year-old male patient was admitted to our emergency department (ED) with diffuse abdominal pain, shortness of breath, cold sweat and palpitations.

On physical examination the patient, with a BMI of 30.7, presented mottled skin of umbilical region and lower extremities; femoral pulses were symmetrically present, while distal ones were absent and he referred leg pain. He was a heavy smoker, without medical history.

Vital signs were as follows: body temperature 36.5°C, blood pressure 100/60 mm Hg, heart rate 120 beats/min and polypnea.

Laboratory analysis (Table 1) revealed leukocytosis, impairment of renal function, increase of D-Dimer, fibrinogen, cardiac troponin-T, C-reactive protein (CRP) and hyperglycemia (with a consequent diagnosis of a previously unknown diabetes). Arterial blood gas (ABG) analysis revealed a compensated metabolic acidosis with respiratory alkalosis and high levels of lactates.

**Table 1.** Results of Laboratory Examinations

Test	Patient’s value	Normal range
White blood cell count (x 10^9^/L)	22,07	4,8–10,8
Hemoglobin (g/dL)	15,2	13,5–17,5
Platelets count (x 10^9^/L)	248	130–400
Creatinine (mg/dL)	2,05	0,72–1,18
Glucose (mg/dL)	327	70–110
Urea (mg/dL)	47	15–50
Sodium	139	135–145
Potassium	4,4	3,30–5,10
Cardiac troponin-T (ng/L)	209	0–14,0
Alanine aminotransferase (U/L)	21	9–59
Creatine kinase (U/L)	89	38–174
D-Dimer (μg/L)	55556	<500
Fibrinogen (mg/dL)	401	165–350
INR	1,12	0,84–1,20
aPTT ratio	0,96	0,86–1,20
C-reactive protein (mg/dL)	2,53	<0,5
Procalcitonin (μg/L)	0,14	0,02–0,06
Ferritin (μg/L)	1069	30–400

The patient underwent a bedside ultrasound in the ED, which showed a large abdominal aortic aneurysm (AAA), without clear signs of rupture. Therefore, a contrast-enhanced multi-detector CT scan was immediately requested.

A multi-phases thoraco-abdominal CT with contrast injection (120 ml of Iopamidol 370 mg ml^−1^ at 4 mL/s) was performed using a 128-slice Flash Dual-Source CT scanner (SOMATOM Definition Flash - Siemens Healthcare). A bolus tracking in the descending aorta, with an attenuation threshold of 120 HU, was used to optimize the imaging of the aorta; an arterial phase with a 10 s delay after reaching the threshold and a venous phase (60 s delay) were obtained. Images were reconstructed with 3 mm and 1 mm collimation. Post-processing included multiplanar reformation (MPR), maximum intensity projection (MIP) and volume rendering technique (VRT) reconstructions.

**Figure 1.**
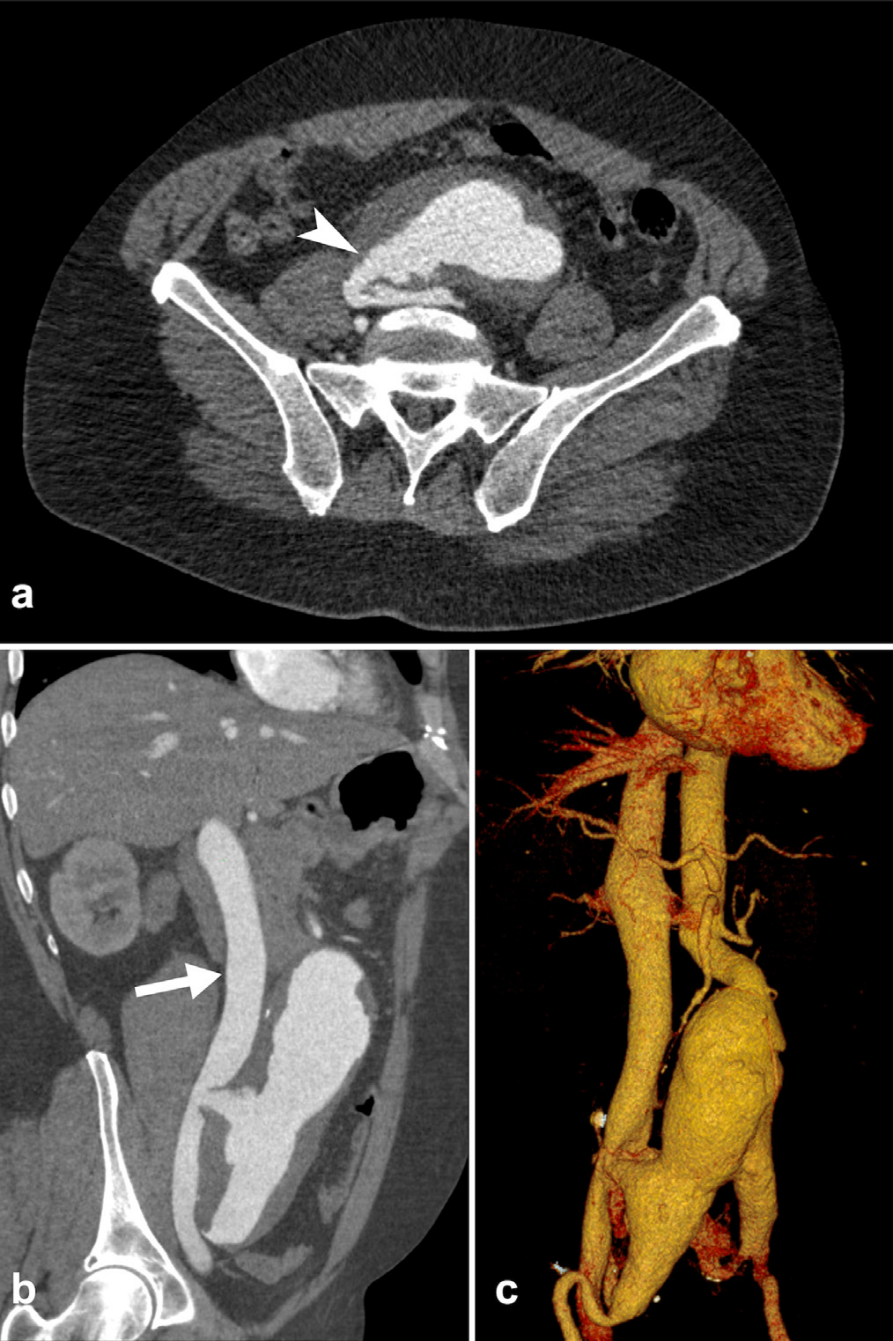
Contrast-enhanced CT scan during arterial-phase showing the presence of a fistula between the aortic aneurysm and the IVC. (**a**) Axial images well depict the communication between the two vessels (arrowhead). (**b, c**) MPR and VRT reformatted images show the early full enhancement of the dilated IVC (arrow) during the arterial phase

Images acquired during the arterial phase showed early full-enhancement of the inferior vena cava (IVC) and hepatic veins associated with a large aorto-bisiliac aneurysm (maximum diameter 8 cm at the infrarenal tract), extended into the iliac bifurcation bilaterally. On axial images, an aortocaval fistula was identified, confirmed on MPR reconstructions, located just above the level of the confluence of the iliac veins. No signs of retroperitoneal rupture of AAA were detected (Figure 1).

**Figure 2.**
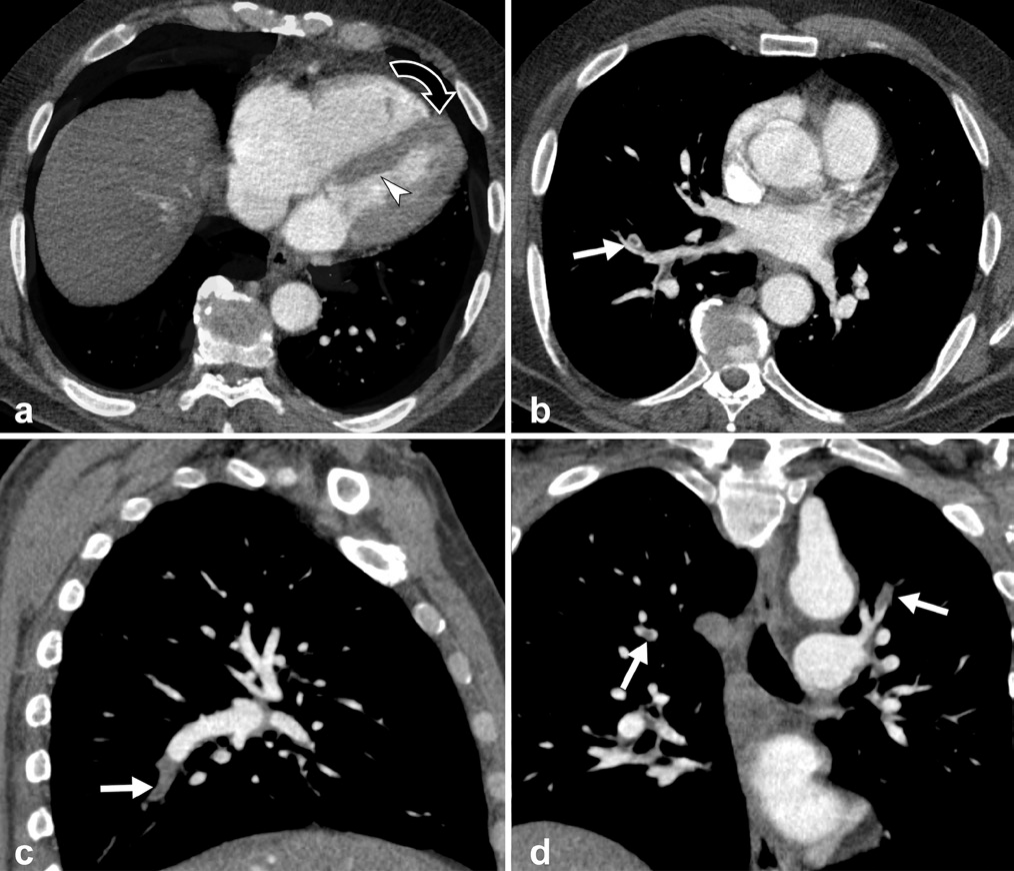
Contrast-enhanced CT scan during arterial-phase. (**a**) Lower chest CT image demonstrates CT signs of right heart overload: enlargement of right atrium and ventricle, cardiac axis clockwise rotation (curved arrow) and straightened interventricular septum (arrowhead). (**b, c, d**) Axial and MPR reformatted images showing bilateral segmental and subsegmental pulmonary embolism (arrows)

AAA was characterized by concentric thrombotic apposition (thickness 20 mm) with adequate proximal neck. Other findings included dilatation of the IVC (infrarenal caliber 27 mm) and CT signs of right heart overload: atrial and ventricle dilatation, cardiac axis clockwise rotation, straightened interventricular septum (Figure 2a).

Last but not least, bilateral acute pulmonary embolism was detected, without CT signs of parenchymal infarctions. Segmental and subsegmental arteries were involved with often complete filling defect (Figure 2b, c and d); main and lobar arteries were patent. The main pulmonary artery presented normal caliber with ascending thoracic aorta / pulmonary artery ratio >1.

After a prompt multidisciplinary evaluation, a surgical approach was chosen, mainly due to a lack of bilateral distal neck necessary for an endovascular treatment.

The patient was submitted to open repair. After median laparotomy, aorta, iliac arteries, IVC and right common femoral vein were isolated to obtain proximal and distal bleeding control. Systemic heparinization with 5000 UI was performed. Aorta and iliac arteries were clamped before sac’s incision.

**Figure 3.**
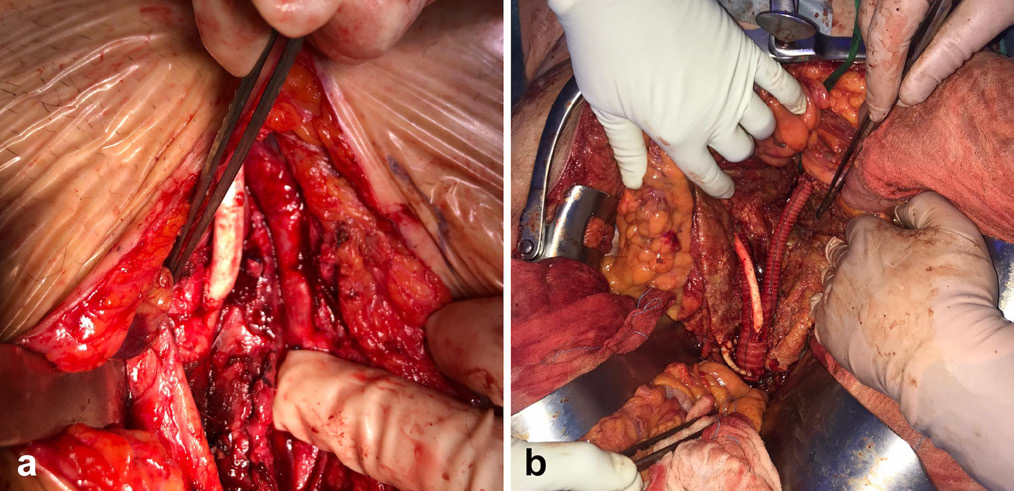
(**a**) IVC suture at the level of aortocaval fistula. (**b**) Aorto-bisiliac reconstruction with Dacron ^®^ prosthesis

A 2 cm fistula between IVC and aorta aortocaval fistula (ACF) was identified and repaired using continues suture with 3/0 Prolene ^®^. Subsequently, an aorto-bisiliac reconstruction with Dacron ^®^ prosthesis (20 × 10 mm) was performed; right hypogastric artery was closed while the left one was saved using Gore^®^ vascular graft (Hybrid nr. 7, Flagstaff, Arizona, USA) (Figure 3).

At the end of the procedure a good refill of IVC, without apparent stenosis, and a valid hypogastric pulsation were obtained.

## Discussion

The rupture of an AAA into the inferior vena cava with fistula formation is a rare condition, occurring in less than 1% of all AAA and in 0.3–11.3% of ruptured AAA, but is associated with a high mortality rate.^1^

The most common site of the ACF is the inferior vena cava, with iliac veins being rarely affected. ACF may coexist with a retroperitoneal rupture or may be isolated, as in our case.^2^ Less common causes of spontaneous ACF include mycotic aneurysm, syphilis, and disorders of connective tissue, such as the Ehlers–Danlos and Marfan syndromes.^3^

The presence of an ACF determines a diversion of blood flow from the high-resistance arterial circuit to the low-resistance venous circuit. This produces a decrease in total peripheral resistance and an increase in venous resistance, pressure and volume, causing right heart overload and high-output cardiac failure.^1,4^

To our knowledge very few cases of pulmonary embolism associated with ACF have been so far described in the literature. Pulmonary embolism may originate from dislodgement of thrombotic material from the AAA in the IVC through the ACF (“paradoxical” embolism). Another source may be the presence of clots developed from venous stasis due to cava compression from the aortic aneurysm.^1,4,5^

Clinical symptoms and findings vary in a wide range and early diagnosis and intervention can be lifesaving. The classic clinical triad of ACF, although not always present, is: abdominal or low back pain, palpable aneurysm and abdominal bruit. Other clinical signs may be present and most likely related to the arterio-venous shunt.^1,3,6^

In our case, patient presented with abdominal pain and clinical sign of a palpable abdominal mass. The abdominal bruit was retrospectively investigated after the CT and reported positive. He also presented shortness of breath, palpitations, leg pain due to peripheral ischemia, and marbled legs related to venous hypertension.

Diagnosis of ACF may be challenging for clinicians, so a prompt emergent imaging is mandatory.

Typical CT findings of ACF include early detection of the contrast medium in the IVC and occasionally the direct visualization of the pathological shunt between abdominal aorta and IVC or iliac veins.^1,7,8^

In our case, CT diagnosis was immediately established due to the early full enhancement of the dilated IVC with direct visualization of the fistula. Bilateral segmental and subsegmental pulmonary embolism was observed.

Our country is currently frontline in the management of the novel Coronavirus pandemic, therefore, ruling out a potential relationship between pulmonary embolism and SARS-CoV2 infection was necessary.^9^ Although no CT findings suggestive for COVID-19 pneumonia were present, and although the patient did not present any other pulmonary symptoms suggestive for COVID-19, a RT-PCR testing on intra operative broncho-alveolar lavage (BAL) was performed and SARS-CoV-2 infection was excluded.

As a result, pulmonary embolism was attributed to a dislodgement of the mural thrombus through the fistula into the IVC (paradoxical). Even if infrequent, our case highlights the necessity to exclude pulmonary embolism in all patients with ACF, especially when AAA presents with a large thrombotic apposition.

Therefore, in the immediate post-operative period, a major problem is represented by the management of anticoagulant therapy to prevent pulmonary embolism.

After the intra-operative administration of 5000 IU Low Molecular Weight Heparin (LMWH), the patient was managed with 4000 IU LMWH twice a day for 7 days and then 6000 IU twice a day until his discharge with the indication to continue the treatment until the first post-operative follow-up.

In the current literature, endovascular repair is considered to be preferable than open repair (OR) because of less surgical stress and shorter hospitalization. However, in the present case, OR was chosen as the treatment option due the lack of bilateral distal neck related to a bilateral aneurysmatic involvement of the iliac bifurcation. Operative treatment includes repair of the fistula with direct suture of the caval defect from inside the aortic lumen and surgical reconstruction of the aorto-iliac axis with a prosthetic graft.^1,6^ In these cases, during follow-up a risk of IVC stricture caused by fibrotic reaction should be considered.

In conclusion, spontaneous ACF caused by aneurysmal rupture is an uncommon condition; its presence can be fatal for potential congestive right heart failure. Since clinical symptoms may be nonspecific, a prompt diagnosis, ideally prior to surgery, is mandatory. In fact, as reported in the literature, intraoperative diagnosis, has a higher mortality rate.^1,3,5^

In most cases instability and severity of symptoms require prompt surgical or endovascular treatment carried out by a skilled team in a well-equipped center.

## Learning points

- Aortocaval fistula (ACF) is a rare complication of abdominal aortic aneurysm and its presence can be fatal for potential right heart failure.
- In presence of an ACF, the dislodgment of thrombotic material from the aorta in the inferior vena cava can lead to paradoxical pulmonary embolism.
- CT angiography can detect the ACF and the pulmonary embolism, leading to a prompt pre-operative diagnosis.
